# Impact of premature birth on cardiopulmonary function in later life

**DOI:** 10.1007/s00431-023-04952-y

**Published:** 2023-05-06

**Authors:** Annika Weigelt, Steffen Bleck, Matthias Jens Huebner, Kathrin Rottermann, Wolfgang Waellisch, Patrick Morhart, Tariq Abu-Tair, Sven Dittrich, Isabelle Schoeffl

**Affiliations:** 1 Department of Pediatric Cardiology, Friedrich-Alexander-Universität Erlangen-Nürnberg, Loschgestrasse 15, 91054 Erlangen, Germany; 2 Department of Neonatology and Intensive Medical Care, Friedrich-Alexander-Universität Erlangen-Nürnberg, Loschgestrasse 15, 91054 Erlangen, Germany; 3 School of Clinical and Applied Sciences, Leeds Beckett University, Leeds, LS13HE UK

**Keywords:** Cardiopulmonary exercise testing, Premature birth, Sports, Habitual exercise

## Abstract

Pulmonary function is reduced in children after preterm birth. The variety of subgroups ranges from early to late preterm births. Limitations in pulmonary function can be observed even after late preterm birth without signs of bronchopulmonary dysplasia and/or history of mechanical ventilation. Whether this reduction in lung function is reflected in the cardiopulmonary capacity of these children is unclear. This study aims to investigate the impact of moderate to late premature birth on cardiopulmonary function. Cardiopulmonary exercise testing on a treadmill was performed by 33 former preterm infants between 8 and 10 years of age who were born between 32 + 0 and 36 + 6 weeks of gestation and compared with a control group of 19 children born in term of comparable age and sex. The former preterm children achieved comparable results to the term-born controls with respect to most of the cardiopulmonary exercise parameters $(\dot{V}O_2peak 43.9\pm6.6\frac{\frac{ml}{kg}}{min}\min vs.41.9\pm8.8\frac{ml}{kg}/\min)$. The only differences were in a slightly higher oxygen uptake efficiency slope $(OUES\;of\;1.6\;\pm0.4\;vs\;1.4\;\pm\;0.4)$ and higher peak minute ventilation $\dot VEpeak\;of\;55.2\pm 11.3 ml/min\;vs.\;49.1\;ml\;\pm\;8.8/min)$ in the group of children born preterm. With respect to heart rate recovery $(-35.3\;\pm\;13.8\;bpm\;vs.\;-37.2\;bpm\;\pm\;14.0\;after\;1\;min)$ and breathing efficiency $\dot{(V}E/\dot VCO_2\;of\;35.9\;\pm\;4.1\;vs\;34.0\;\pm4.6)$, there were no significant differences.

*Conclusion*: Children born preterm did not show limitations in cardiopulmonary function in comparison with matched controls.

**Table:** 

**What is Known:** • *Preterm birth is associated with reduced pulmonary function in later life, this is also true for former late preterms.* • *As a consequence of being born premature, the lungs have not finished their important embryological development. Cardiopulmonary fitness is an important parameter for overall mortality and morbidity in children and adults and a good pulmonary function is therefore paramount.*	
**What is New:** • *Children born prematurely were comparable to an age- and sex-matched control group with regards to almost all cardiopulmonary exercise variables.* • *A significantly higher OUES, a surrogate parameter for VO*_2_*peak was found for the group of former preterm children, most likely reflecting on more physical exercise in this group. Importantly, there were no signs of impaired cardiopulmonary function in the group of former preterm children.*

**What is Known:**

• *Preterm birth is associated with reduced pulmonary function in later life, this is also true for former late preterms.*

• *As a consequence of being born premature, the lungs have not finished their important embryological development. Cardiopulmonary fitness is an important parameter for overall mortality and morbidity in children and adults and a good pulmonary function is therefore paramount.*

**What is New:**

• *Children born prematurely were comparable to an age- and sex-matched control group with regards to almost all cardiopulmonary exercise variables.*

• *A significantly higher OUES, a surrogate parameter for VO*_2_*peak was found for the group of former preterm children, most likely reflecting on more physical exercise in this group. Importantly, there were no signs of impaired cardiopulmonary function in the group of former preterm children.*

## Introduction

In industrialized countries, the rate of preterm births has risen in recent years with the largest proportion of these being late preterm births [1]. Even though most studies investigating long-term health and disease after preterm birth are mainly limited to children born extremely (< 28 weeks) and very (28–32 weeks) preterm, recent reports show adverse long-term outcomes also in children born moderately (32–37 weeks) preterm [2, 3]. In a recent large-scale study, investigating the mortality of young adults born preterm and early term across four Nordic nations in increased death risk was found across gestational ages [2]. In this study, the excess mortality was most pronounced for cardiovascular deaths, chronic lung disease, and diabetes [2]. It has also been observed that children born preterm could have a reduced ability to exercise due to pulmonary and neurological deficits [4–7]. This reduced ability to exercise can be measured through the peak oxygen uptake ($\dot{V}{O}_{2}peak)$ during exercise. The parameter reflects the amount of oxygen available for muscle consumption and equals the product of cardiac output and arterio-venous oxygen difference [8]. Thus, the $\dot{V}{O}_{2}peak$ depends on the pulmonary, cardiovascular, and muscular systems and increases with increasing workload.

Cardiorespiratory fitness (CRF) is considered to be the most important marker of health among the health-related physical fitness components in children and adolescents [9, 10]. The association between levels of CRF during childhood and cardiovascular disease risk factors later in life are inverse, making it so much more important to increase CRF early and to distinguish the causes for a reduced capacity early on. In a systematic review, Edwards et al. [7] were able to show that a former premature birth leads to a lower $\dot{V}{O}_{2}peak$ in later life when compared to term-born controls, though the difference is small.

When investigating the effects of premature birth on $\dot{V}{O}_{2}peak$, the components of this parameter need to looked at in turn, i.e., pulmonary, cardiac and motor function.

The lung as well as the airway is being formed by the 16th week of gestational age, but true alveoli do not begin to develop until about 30th week, with subsequent increase in number, size, and complexity during the first 3–4 years of life [11, 12]. As a consequence of preterm delivery, lung development that would normally have taken place in utero will then occur post-natal instead but under very different conditions [12]. These include active breathing with strain, distension of immature lung tissue, and lung perfusion with full cardiac output. All this happens while being exposed to considerably higher oxygen tension than during fetal life [12]. As the long-term survival of preterm infants increases, long-term lung recovery and health become more important.

Apparently, moderate to late preterm birth (32–37 weeks of gestation) leads to the interruption of a critical period of rapid in utero respiratory growth [13–16]. The deviation from the finely programmed series of normal lung development may be responsible for alterations in pulmonary mechanics during infancy. This in turn can lead to an overly compliant chest wall, reduced expiratory airflow, and increased airway resistance at birth [15, 16].

Several studies have investigated the effects of moderate prematurity on pulmonary function using classic lung function tests. Compared with their peers born at term, these infants and toddlers show an increased respiratory morbidity [17–19]. Especially for late preterm infants who did not show signs of bronchopulmonary dysplasia and who did not experience mechanical ventilation because of their premature birth, these findings are surprising [5, 14, 20, 21]. The question arises whether these findings translate into a true impairment of cardiopulmonary function in the daily life of former preterm infants.

Apart from affecting lung development, there is evidence that prematurity affects the development of the autonomic nervous system (ANS) as this develops in the third trimester of pregnancy [22] and is disrupted by preterm birth. Consequently, preterm infants but also children and young adults exhibit a lower heart rate variability with a delayed heart rate recovery (HRR) [23, 24]. Such a decreased HRR represents a negative predictor for cardiovascular disease and all-cause mortality [25, 26]. In a study investigating the effect of early preterm birth on HRR in young adults, several studies were able to prove such a slower HRR compared to term-born controls and thus concludes that these children may be at higher risk for developing cardiovascular disease in later life [23, 27].

Another explanation for the reduction in HRR is provided by Huckstep et al. [27] who suggest that myocardial impairment leads to an insufficient increase in cardiac contractility to maintain adequate muscle perfusion. The ensuing exaggerated peripheral vasoconstriction to sustain pressure increases systemic vascular resistance, increases afterload, and further impairs stroke volume. The ensuing hypoperfusion of metabolite eliminating organs leads to a delayed clearance and deactivation of the metaboreflex which controls HRR (Michael 2017). The authors explain the observed reduction in exercise capacity and HRR in their late preterm group with a reduced stroke volume response to physical exercise using echocardiography at 40% and 60% work intensity [27].

The study of the muscular performance of former preterm children is mainly limited to children born very preterm or of extremely low birthweight [28–30]. The motor deficits that were observed in these studies include poor coordination and deficits in postural stability [28–30]. When investigating the cardiopulmonary fitness of these children using a 20-m shuttle run test alongside their motor coordination, the most powerful predictor for $\dot{V}{O}_{2}peak$ was not the respiratory function but the results from the Movement Assessment Battery for Children (MABC) [30].

How important then is the effect of prematurity on cardiopulmonary exercise capacity and its consequences in later life? Can the increased lifelong risk for cardiovascular and pulmonary mortality be predicted by cardiopulmonary exercise testing? Is the saying true “once a preterm always a preterm” or is it possible to achieve good cardiopulmonary function even after being born preterm?

In order to address these questions, we wanted to investigate the implications of a late preterm birth (32–37 weeks of gestation) on the cardiopulmonary exercise performance at the age of 8–10 years of age using cardiopulmonary exercise test (CPET) on a treadmill which is best suited to identify differences in $\dot{V}{O}_{2}peak$ in former preterm children [7]. The main objective was to compare peak oxygen uptake, and the secondary objective was to identify the respective influence of the cardiac and pulmonary parameters measured during CPET on the cardiopulmonary function of former moderately preterm born children.

## Material and methods

The study was approved by the Ethics Committee of the University of Erlangen-Nuremberg, FRG (35_18B). All study participants as well as their legal guardians gave written informed consent according to the standards set by the Declaration of Helsinki.

### Participants

Every child treated at our university hospital is recorded in the hospital database along with its diagnosis. The list of all children born moderately to late preterm (32 + 0 to 36 + 6 weeks of gestation) in the age range of 8–10 years was then scanned for additional pathologies using the respective discharge letters. Every child with an additional pathology apart from being preterm was excluded from the study. Children who had received mechanical ventilation requiring an intubation or CPAP for more than 24 h were also excluded from the study. Children who had received oxygen for more than 28 days were also excluded from the study in order to eliminate children with signs of bronchopulmonary dysplasia [31].

Thus, the inclusion criteria were as follows:

- 32 + 0 – 36 + 6 weeks’ gestation
- 8–10 years of age at the time of the examination (born between 2010 and 2012)
- No mechanical ventilation
- At time of discharge no clinical signs of bronchopulmonary dysplasia
- No additional pathologies

The control group consisted of otherwise healthy children of the same age and gender (born in the same years), with normal birthweight. Inclusion criteria for all participants were the ability to complete an exercise test, be free of mental, physical, or neurological disabilities and had no diagnosed cardiovascular or respiratory disease. The recruitment was performed among the children of staff and friends who fitted the inclusion criteria.

Height and weight were measured using a stadiometer and electronic scale (Seca 704 S, Hamburg, Germany).

Physical activity was assessed by a general questionnaire previously described [32], in hours per week. This questionnaire was filled out by the parents of the child. Medical records from the preterm group were consulted for the inclusion criteria. Tidal volume as an index of respiratory function was recorded prior to CPET.

### Measurement of Gas exchange

A small, low-dead-space respiratory valve (88 ml) with a size-matched mouthpiece and headgear was used (Metalyzer 3B, Cortex, Leipzig, Germany). During each test, the gas-exchange was measured continuously using a breath-by-breath method and averaged over 15-s intervals. We used the following physiological criteria for completion of a valid $\dot{V}{O}_{2}peak$, two of which needed to be met for validation: (1) peak heart rate (peak HR) within 5% of the age-predicted maximum, (2) respiratory exchange ratio (RER) ≥ 1.0, and (3) volitional fatigue [32, 33]. We chose a threshold of 1.0 RER for completion of a valid $\dot{V}{O}_{2}peak$ as it is difficult to achieve higher RER values when performing CPET on a treadmill with children [34].

We used the V-slope method proposed by Beaver et al. [35] to determine the ventilatory thresholds VT_1_ and VT_2_. Plotting $\mathrm{oxygen uptake} (\dot{V}{O}_{2)}$ (ml/min) against the logarithm of minute ventilation (VE) (ml/min) and calculating the slope of this linear relation through single regression analysis [32] determined oxygen uptake efficiency slope (OUES).

### Cardiopulmonary exercise test

Any acute disease was outruled by a physical exam of each subject before the cardiopulmonary exercise test.

All subjects were fitted with a heart rate (HR) monitor (Polar H7 Bluetooth Smart 4.0 heart rate sensor, Kempele, Finland) as well as a 12-lead ECG (Custo®) for monitoring heart rate and ECG changes. Expired gases were collected breath-by-breath (Metalyzer, Cortex, Germany), and HR, ventilatory, and metabolic parameters were recorded and analyzed in the Metasoft Studio (Cortex, Germany). The ventilatory threshold 1 (VT_1_) was determined using the V-slope method proposed by Wasserman [36]. A 20-s rolling average was used to determine ventilatory thresholds and $\dot{V}{O}_{2}peak$. The $\dot{V}E/\dot{V}C{O}_{2}$-slope as the slope of the relationship between $\dot{V}E$ and carbon dioxide elimination $\dot{(\mathrm{V}}C{O}_{2}$) was determined between the beginning of each test and the first ventilatory threshold VT_1_ [37]. OUES was determined by the slope of $\dot{V}{O}_{2}$ against the logarithm of $\dot{V}E$ between the beginning of the exercise and VT_1_ [38].

Spirometry was performed before each test using the cardiopulmonary exercise equipment, in order to allow for the estimation of the breathing reserve. However, since the data was not obtained using a bodyplethysmograph, we do not trust it to be accurate and therefore do not present it in this article.

An incremental step test on a treadmill (COSMED T 170, COSMED, Italy) was performed for cardiopulmonary exercise testing. We used an age-appropriate treadmill testing protocol derived from a previous study [32]. In this protocol, the starting speed is set at 3 km/h, with the following steps set at 6 km/h, 8 km/h, and then an increase of 1 km/h every 2 min. We used an increment of 1% for simulation of a natural environment. All participants were encouraged verbally to run until exhaustion and all tests were performed by the same researchers. The researchers undertaking the CPET were all trained in the execution of these tests. They were not blinded to neonatal information.

### Statistical analysis

Statistical analysis was performed using Microsoft Excel 2000® for data collection and SPSS 12.0® (SPSS Inc., Chicago, IL) for statistical evaluation. All measured values are reported as means and standard deviations. The Kolmogorov–Smirnov test was used to check for normal distribution. Homogeneity of variance was investigated using Levene’s *F*-test. For normally distributed variables, differences between the former preterm children and their healthy control group were assessed with unpaired *t*-tests; otherwise, the Wilcoxon or the Whitney-Mann *U*-tests were used. Statistical significance was set at *p* < 0.05.

We did not perform a power analysis as we planned to recruit all eligible subjects who were willing to participate.

## Results

### Subjects

We tested 33 former preterm born children after 32 + 0 weeks of gestation (17 male and 16 female) and matched these subjects to 19 at term-born children of comparable age and sex (10 male and 9 female). Pre- and postnatal descriptors of the preterm group are presented in Table 1. The anthropometric data as well as the amount of physical activity performed by the children determined using the physical activity questionnaire are depicted in Table 2. There were no significant differences between the two groups regarding age, height, weight, BMI, or amount of physical activity.

**Table 1:** Frequency of pre- and postnatal parameters as well as means and standard deviation of gestational age, the age of the mother at delivery, and birthweight of the late preterm group

	**Distribution**
Gestational age (weeks)	34.9 ± 1.9
Birthweight (g)	2350 ± 470.5
Age mother (years)	32.5 ± 5.8
Tobacco use of the mother during pregnancy	11.8%
Prenatal steroids given to the mother before birth	26.5%
Cesarean section	73.5%
Cpap therapy after birth	14.3%
RSV prophylaxis	8.3%

CPAP continuous positive airway pressure, RSV respiratory syncytial viruses

**Table 2:** Anthropometric data as well as extracurricular sports participation as means and standard deviation (*signifies a significant difference between the two groups)

	**Preterm (33)**	**Term-born (19)**	***p* Value**
Age (years)	9.2 ± 0.6	8.8 ± 1.1	0.10
Weight (kg)	33.0 ± 8.6	30.3 ± 5.3	0.23
Length (cm)	140.1 ± 10.3	137.5 ± 9.5	0.36
BMI (kg/m^2^)	16.5 ± 2.6	15.9 ± 1.4	0.34
Females (*n*)	16 (48.5%)	9 (52.6%)	0.6
Males (*n*)	17 (51.5%)	10 (47.4%)	0.6
Sport performed in childhood (hours/week)	3.3 ± 5.5	3.3 ± 2.1	0.98
Sport performed at school age (hours/week)	4.2 ± 4.6	3.4 ± 1.8	0.69

Clarifying definition: childhood, < 6 years; at school age, ≥ 6 years

### Aerobic exercise capacity

Results from the cardiopulmonary exercise test are represented in Table 3. Both groups achieved comparable maximal effort with peak RER above 1.0 reflecting on the good participation of the subjects. The time to achieve $\dot{V}{O}_{2}peak$ (exercise-time) was well within the timeframe of 7–11 min for achieving peak exertion allowing for a good comparability of the tests. There were no significant differences for exercise times or maximum speed achieved between the two groups.

**Table 3:** CPET values as means ± standard deviation assessed with an unpaired *t*-test (asterisk (*) identifies a statistical significance set at *p* < 0.05)

	**Preterm**	**Term-born**	***p* Value**
RER	1.06 ± 0.05	1.05 ± 0.07	0.61
Peak speed (km/h)	10.3 ± 1.1	9.8 ± 1.4	0.20
Peak HR (bpm)	193.0 ± 11.0	192.3 ± 8.9	0.81
VO_2_peak (ml/min/kg)	43.9 ± 6.6	41.9 ± 8.8	0.36
VEpeak (ml/min)	55.2 ± 11.3	49.1 ± 11.1	0.07
BR (breaths/min)	66.0 ± 9.2	68.9 ± 10.3	0.31
Breathing reserve (ml/min)	15.9 ± 20.2	27.4 ± 24.1	0.08
O_2_puls (ml/min)	7.2 ± 1.6	6.6 ± 1.3	0.14
OUES*	1.6 ± 0.4	1.4 ± 0.4	0.02
VE/VCO_2_	35.9 ± 4.1	34.0 ± 4.6	0.14
HRR after 1 min (bpm)	− 35.3 ± 13.8	− 37.2 ± 14.0	0.63
HRR after 2 min	− 52.9 ± 11.2	− 51.5 ± 10.8	0.67

RER respiratory exchange ratio at the point of maximal exertion, peak HR peak heart rate, VO2peak peak oxygen uptake, VEpeak minute ventilation at peak exercise, BR breathing rate, OUES oxygen uptake efficiency slope, VE/VCO2-slope correlation between expiratory volume to the volume of CO2, HRR heart rate reserve

**Fig. 1:**
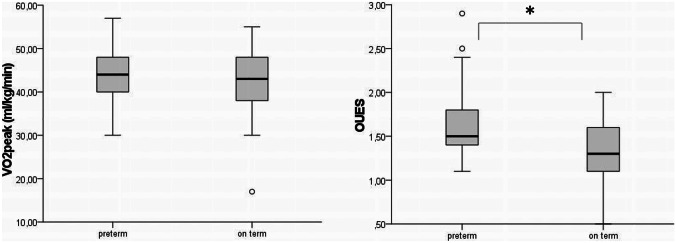
$\dot{\mathrm{V}}{\mathrm{O}}_{2}\mathrm{peak}$ and OUES in former preterms and term-born children. Median, interquartile range, and minimum and maximum of $\dot{\mathrm{V}}{\mathrm{O}}_{2}\mathrm{peak}$ (ml/kg/min) and OUES determined in the group of former preterms in comparison with the group of term-born children. Abbreviations: VO2peak, peak oxygen uptake; OUES, oxygen uptake efficiency slope

The $\dot{V}{O}_{2}peak$ did not differ significantly between the two groups. However, the OUES, a surrogate parameter of aerobic exercise capacity at submaximal exercise (s. Fig. 1), was significantly higher in the former preterm group.

The heart rate at peak exercise was comparable between former preterm children and their age-matched control group (s. Table 3).

### Pulmonary function

**Fig. 2:**
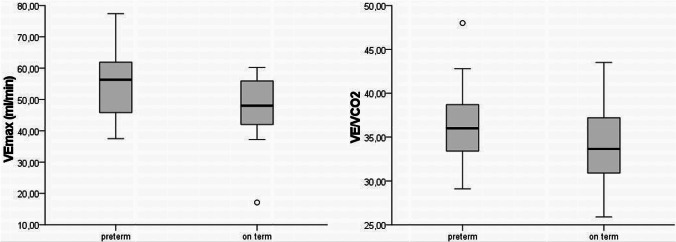
$\dot{\mathrm{V}}\mathrm{Epeak}$ and $\dot{\mathrm{V}}\mathrm{E}/\dot{\mathrm{V}}{\mathrm{CO}}_{2}$ in former preterms and term-born children. Median, interquartile range, and minimum and maximum of $\dot{\mathrm{V}}\mathrm{Epeak}$ (ml/kg/min) and $\dot{\mathrm{V}}\mathrm{E}/\dot{\mathrm{V}}{\mathrm{CO}}_{2}$ determined in the group of former preterms in comparison with the group of term-born children. Abbreviations: VEpeak, minute ventilation at peak exercise; VE/VCO2-slope, correlation between expiratory volume to the volume of CO2

Even though the breathing frequency did not differ between the two preterm groups, the peak minute ventilation $\dot{(V}Epeak)$ was slightly higher in the group of preterms compared to the control group (s. Table 2) without reaching significance. The breathing rate (BR) was comparable between the two groups, but the breathing reserve was lower in the preterm group. These differences did not reach significance. The ratio of $\dot{V}E/\dot{V}C{O}_{2}$ is a representative parameter for respiratory efficiency. Even though the former preterm children exhibited higher values than their term-born counterparts, this difference did not reach level of significance (s. Fig. 2).

### Cardiac function

The cardiac output can be determined in cardiopulmonary exercise by applying the Fick equation and calculating the O_2_pulse. In this study, the former preterm children achieved higher values for O_2_pulse, but this difference did not reach significance (s. Table 3). The $\dot{V}E/\dot{V}C{O}_{2}$ slope evaluates the mismatch between ventilation and perfusion. Values believed to be consistent with heart failures are above 30. In this study, both groups showed values above 30 (36 in former preterms and 34.1 for former term-borns) as represented in Table 3.

HRR allows to evaluate the autonomic nervous system. In this study the HRR 1 and 2 min after termination of exercise was comparable between the two groups (s. Table 3).

## Discussion

The former moderately born preterm children in this study did not exhibit any limitation in exercise capacity. Previously observed limitations with respect to pulmonary and/or cardiac function could not be verified in this study. Whether physical activity could have helped overcome any cardiopulmonary disadvantages cannot be deduced from this retrospective study, but we still believe that moderate to late preterm birth does not necessarily involve cardiopulmonary consequences if these children participate in physical activity on a regular basis.

Interestingly, in this study, the cardiopulmonary function, represented by $\dot VO_2peak$, did not differ significantly between children preterm and those born at term. A comparable $\dot{V}{O}_{2}peak$ between children and young adults born preterm and their term-born counterparts has previously been observed in other studies [7, 39, 40], even including children born extremely preterm [41]. These results stand in contrast to other studies which showed significantly reduced $\dot{V}{O}_{2}peak$ in preterm-born children and young adults [23, 27, 42]. However, a meta-analysis on the effect of preterm birth on exercise capacity concluded that even though the $\dot{V}{O}_{2}peak$ of preterm children is lower than in term-born control subjects, this difference is usually small and they are able to achieve near normal exercise capacity [7]. The values for $\dot{V}{O}_{2}peak$ achieved in our study by both groups were well comparable to previous studies of this parameter in children born preterm using the treadmill for CPET [7].

Since we chose a rather low threshold for RER for defining peak exertion (RER > 1.0), the values for $\dot{V}{O}_{2}peak$ might have been higher with a more suitable testing method like testing outdoors [34, 43]. However, the OUES is unaffected by peak exercise as it is defined as the slope of the linear relation through single regression analysis of $\mathrm{oxygen}\;\mathrm{up}\;\mathrm{take}\;(\dot VO_{2)}$ (ml/min) plotted against the logarithm of minute ventilation (VE) (ml/min) between the beginning of exercise and VT1.

Consequently, it was surprising that the OUES, a surrogate parameter for exercise function determined at a submaximal level, was significantly higher in the preterm group. As it can be difficult to motivate children to exercise to maximum exhaustion, the OUES presents a unique parameter for estimating exercise function at submaximal exercise [38]. One previous study investigated the OUES differences between preterm and term-born control [40]. In this study, there were no significant differences between the two groups, and they underlined the importance to integrate this parameter in such studies as it is independent of maximal exertion [40]. The fact that the preterm children in this study achieved higher results in the OUES than their term-born controls underlines the fact that children born preterm are not less performant than their term-born counterparts. However, the difference between the two groups was so small that we do not want to suggest that preterm-born children have a higher cardiopulmonary capacity than their term-born counterparts. It simply underlines the fact that they were not less performant than their term-born counterparts.

The fact that former preterm children are not less performant than their term-born counterparts is not that surprising given the relatively mild impairment in pulmonary structure as a consequence of preterm birth [40]. As the respiratory reserve typically exceeds 30% [44], the relatively mild impairment in pulmonary structure due to preterm delivery does not limit aerobic exercise [40]. However, the peak minute ventilation $\dot{V}E$ was significantly higher in the group of preterm children compared to the controls born at term in our study. A previous study investigating $\dot{V}E$ in extremely preterm children found lower values for these children born to control children [45]. Since the children in our study were only former moderately to late preterm children, this could explain the difference of these results. Still, it means that children born even moderately to late preterm compensate with a higher $\dot{V}E$ to achieve a comparable $\dot{V}{O}_{2}peak$. Another parameter to consider with respect to ventilatory limitations is the slope of $\dot{V}E/\dot{V}C{O}_{2}$, which indicates increased dead space ventilation when increased [46]. This in turn could be related to increased alveolar dead space, or increased anatomical dead space ventilation due to different breathing patterns when breathing frequency is increased [46]. However, neither the $\dot{V}E/\dot{V}C{O}_{2}$ slope nor the breathing frequency differed significantly between the two groups.

Elevation in the $\dot{V}E/\dot{V}C{O}_{2}$ slope can also be observed secondary to deficits in cardiac performance, which drive a compensatory response of increased ventilation [27]. The phenomenon of an increased $\dot{V}E/\dot{V}C{O}_{2}$ slope can often be observed in patients with heart failure where the values can often exceed 30 and more [27]. Decreased cardiac output, a sign of myocardial impairment as a reason for decreased lower exercise capacity, has previously been observed in adolescents born very preterm using thoracic bioimpedance during exercise testing [39]. Another study reported an association of preterm birth with ischemic heart disease in adults born moderately preterm [47]. A recent study determined the ejection fraction (EF) using exercise echocardiography at rest, at 40% and 60% of CPET peak power [27] in order to estimate myocardial functional reserve. They were able to prove a correlation of the EF with percent of predicted $\dot{V}{O}_{2}peak$ which was reduced in the young adults born moderately preterm [27]. In our study, peak cardiac output determined by using the O_2_ pulse as surrogate parameter, was comparable between the two groups. So, even though the $\dot{V}E/\dot{V}C{O}_{2}$ slope was slightly higher in the group of preterm infants, this possible diffusion/perfusion-mismatch of the lung due to reduced cardiac function could not be objectified in our study. We also observed slightly increased values for the $\dot{V}E/\dot{V}C{O}_{2}$ slope across the preterm- and term-born children. Further studies are needed in children in order to determine whether children tend to have higher values for this slope as this increase was observable across all children tested in this study.

Another sign of myocardial impairment is a delayed HRR [27]. Myocardial impairment results in hypoperfusion of metabolite eliminating organs, causing a delay in the clearance and deactivation of the metaboreflex [27] which leads to earlier fatigue and a delayed HRR [48]. Another explanation for a delayed heart rate recovery is an impairment of ANS development as this develops significantly during the third trimester [22]. HRR after maximal exercise is a consequence of the reactivation of the parasympathetic nervous system and sympathetic withdrawal [49] and is recognized as a marker of autonomic function [50]. A deficient vagal tone (parasympathetic regulation) is associated with all-cause mortality and cardiovascular disease in later life [51], and young adult survivors of prematurity have been observed to develop more likely hypertension [52] and right and left ventricular hypertrophy along with lower right ventricular ejection fraction compared to term-born controls [53]. In our study, HRR was comparable between the two groups after 1 and 2 min. One possible explanation could be that the majority of participants in our study was late preterm and thus may have been less affected of a delayed development of the ANS [54–58].

The strengths of this study include the high number of moderate preterm-born children that could be recruited for cardiopulmonary exercise testing as well as the matched control group of term-born children. Since all participants were treated at the same institution according to the same standard of practice, there is no bias regarding pre-, peri-, and postnatal treatment. All tests were conducted according to the same protocol using the same CPET equipment allowing for a good comparability between the results. This is also one of the few studies investigating cardiopulmonary function in former preterm children including an evaluation of physical exercise in the studied group. There are several limitations in this study. The results may be biased towards children with better exercise capacity, since the participation was voluntary. Measurements of activity were collected by questionnaire rather than objective measurements. The researchers performing CPET were not blinded to neonatal information which may have led to a bias of the results. Even though the number of tested children was comparably high, it was still limited due to being a single center study.

## Conclusion

We examined moderate preterm born 10 – 12-year-old children and found that their exercise capacity (VO_2 _peak) and HRR were similar to term born controls. Since the preterm-born children in this study performed relatively large amounts of physical exercise and physical exercise is known to increase exercise capacity, further studies are needed for evaluating the effects of physical exercise on the observed increased mortality due to cardiovascular disease, chronic lung disease, and diabetes in young adults born preterm and early term [2].

## Data Availability

Data can be made available upon request.

## Abbreviations

ANS: Autonomic nervous system
BPD: Bronchopulmonary dysplasia
BR: Breathing rate
CPAP: Continuous positive airway pressure
CPET: Cardiopulmonary exercise test
CRF: Cardiorespiratory fitness
HR: Heart rate
HRR: Heart rate recovery
OUES: Oxygen uptake efficiency slope
Peak HR: Peak heart rate
peakRER: RER at maximal exertion
RER: Respiratory exchange ratio
RSV: Respiratory syncytial virus
$\dot{\mathrm{V}}C{O}_{2}$: Carbon dioxide elimination
$$\dot{V}E$$: Minute ventilation
$\dot{V}Epeak$: Peak minute ventilation
$$\dot{V}E/\dot{V}C{O}_{2}$$: Breathing efficiency
$\dot{V}{O}_{2}$: Oxygen up take
$\dot{V}{O}_{2}peak$: Peak oxygen uptake
VT_1_: Ventilatory threshold 1
VT_2_: Ventilatory threshold 2
